# Dynamics of the natural enemy community of *Hyphantria cunea* (Lepidoptera: Erebidae) in Dandong, China

**DOI:** 10.1093/jisesa/iead105

**Published:** 2023-11-28

**Authors:** Xinyang Zhang, Liyuan Yang, Chen Chen, Junrui Shi, Yixin Zhang, Shouhui Sun

**Affiliations:** College of Forestry, Shenyang Agricultural University, Shenyang, China; College of Forestry, Shenyang Agricultural University, Shenyang, China; College of Forestry, Shenyang Agricultural University, Shenyang, China; College of Forestry, Shenyang Agricultural University, Shenyang, China; College of Forestry, Shenyang Agricultural University, Shenyang, China; College of Forestry, Shenyang Agricultural University, Shenyang, China

**Keywords:** invasive forestry pest, predator, parasitoid, dominant species, complexity

## Abstract

This study aims to explore the composition of natural enemy species in the fall webworm, *Hyphantria cunea* (Drury) population and the dynamics of its natural enemy community in Dandong, Liaoning Province, China, where it was first reported. We collected the natural enemy of eggs, larvae, and pupae of *H. cunea* on host trees at 12 survey sites from June 2019 to October 2020. The results showed that the community consists of 34 species: 20 predatory species, including 15 spiders and 5 insects, and 14 parasitic species, including 10 parasitic wasps and 4 parasitic flies. The top 3 dominant species based on the importance value index for both parasitic and predatory species were *Pediobius pupariae* > *Chouioia cunea* > *Cotesia gregalis* in the natural enemy community of *H. cunea*. Analysis of all 3 principal components by principal component analysis showed that Clubionidae sp. 1, *Parena cavipennis*, or other predators were the main factors affecting the natural enemy community. Analysis of the community structure parameters of the *H. cunea* natural enemy community in different developmental stages across generations revealed the following: (i) Compared with the degree of complexity of the egg and pupal stages, the larval stage was the highest. (ii) The complexity was determined by means of comprehensive evaluation: first-generation larvae in 2020 > first-generation larvae in 2019 > second-generation larvae in 2020 > second-generation larvae in 2019. These results clarify the dynamics of natural enemy species, coevolution with the host in the invaded habitat of *H. cunea* and development of biological control technologies.

## Introduction

The fall webworm, *Hyphantria cunea* (Drury) (Lepidoptera: Erebidae), is a serious exotic forestry pest in China (Bi et al. 2018, Li et al. 2020, Sun et al. 2021). It is native to North America and distributed widely in the United States, southern Canada, and northern Mexico (Pimentel et al. 2001). In 1979, this invader was introduced into Dandong, Liaoning Province, it is where the species was first reported in China (Yu 1993, Sullivan et al. 2012, Yu and Li 2015). By 2023, the domestic epidemic area had spread to 14 provinces (autonomous regions, cities) and 611 county-level regions (Tao et al. 2023).

*Hyphantria cunea* has 2–3 generations per year in Dandong, China (Yan et al. 2015). Larvae feed within a silken web, and they cause rampant hazard mainly during the larval stage (Chen 2017). Its larvae emerge in late May (Suzuki et al. 2018). The food consumption of *H.cunea* larvae begins to increase at the 5th instar. It can feed on approximately 600 species of deciduous trees worldwide (Ning et al. 2022).

In North America, *H. cunea* is not considered a problem in forests because it primarily attacks tree species with little economic value (Furniss and Carolin 1977). In China, the preferred host plants include broadleaf trees such as *Morus* spp., *Salix* spp., *Populus* spp., *Robinia pseudoacacia*, and some fruit trees. Sometimes endangered *Metasequoia glyptostroboides*, *Taxodium distichum*, *T. ascendens*, and other coniferous species have been targeted (Mason et al. 2011, Yuan 2020). *Hyphantria cunea* can also harm wheat, soybean, corn, etc. when faced with serious attacks or host tree shortages (Wan 2020, Lu et al. 2021). This pest has expanded its range rapidly and caused unprecedented economic losses due to its omnivorous feeding habits, tolerance to starvation, strong adaptability, insufficient prevention and control methods, and lack of in-depth research on the relevant enemies (Lin et al. 2016, Huang et al. 2018, Sun et al. 2021, Ning et al. 2022).

*Hyphantria cunea* has been infesting trees for more than 4 decades since it was introduced into Dandong, Liaoning Province, China, in 1979. As such, there should be a higher abundance of the natural enemies of *H. cunea* in Dandong, as it is where the species was first introduced in China. Previous research results for natural enemies in different stages of *H. cunea* indicated the following: (i) The main predators of the egg stage are *Chrysopa pallens* (Rambur) (Neuroptera: Chrysopidae), *Chrysoperla carnea* (Stephens) (formerly *Chrysopa shansiensis*; Neuroptera: Chrysopidae), *Chrysopa formosa* Brauer (Neuroptera: Chrysopidae), *Chrysoperla furcifera* (Okamoto) (formerly *Chrysopa kulingensis*; Neuroptera: Chrysopidae), *Harmonia axyridis* (Pallas) (Coleoptera: Coccinellidae), and *Himacerus apterus* (F.) (Hemiptera: Nabidae). (ii) Predators of the larval stage include *Parena cavipennis* (Bates) (Coleoptera: Carabidae), *P. latecincta* (Bates) (Coleoptera: Carabidae), *P. laesipennis* (Bates) (Coleoptera: Carabidae)*, Arma chinensis* Fallou (Hemiptera: Pentatomidae) (Wang et al. 2012, 2021, Chen et al. 2020, Sun et al. 2021), and more than 20 species of spiders (Shu and Yu 1985, Wang et al. 1999, Yang et al. 2008, Sun et al. 2021). In addition, *Bufo gargarizans* Cantor (Anura: Bufonidae), *Pelophylax nigromaculatus* (Hallowell) (Anura: Ranidae), and some common birds can also prey on larvae, such as *Passer montanus* (L.) (Passeriformes: Passeridae), *P. domesticus* (L.) (Passeriformes: Passeridae), *Cyanopica cyanus* (Pallas) (Passeriformes: Corvidae), and *Pycnonotus sinensis* (Gimelin) (Passeriformes: Pycnonotidae). (iii) The pupal stage predators include *Chlaenius pallipes* (Gebler) (Coleoptera: Carabidae), *Nebria livida* (L.) (Coleoptera: Carabidae), ants, and spiders (Shu and Yu 1985, Tao et al. 2008, Zhang et al. 2008, Li 2011, Sun et al. 2021).

There are 49 parasitic species of *H. cunea*, including 36 parasitic wasps and 13 parasitic flies. The main species of parasitoids include *Dolichogenidea singularis* Yang et You, sp. nov (Hymenoptera: Braconidae), *Cotesia gregalis* Yang et Wei (Hymenoptera: Braconidae), *Pimpla disparis* Viereck (Hymenoptera: Ichneumonidae), *Chouioia cunea* Yang (Hymenoptera: Eulophidae), *Pediobius pupariae* Yang (Hymenoptera: Eulophidae), *Eupelmus fulvipes* Förster (Hymenoptera: Chalcididae), and *Pediobius* sp. (Hymenoptera: Chalcididae). The main species of parasitic flies include *Compsilura concinnata* (Meigen) (Diptera: Tachinidae), *Exorista japonica* (Townsend) (Diptera: Tachinidae), *E. fasciata* (Fallén) (Diptera: Tachinidae), *Carcelia kockiana* Townsend (Diptera: Tachinidae), and *Blepharipa zebina* (Walker) (Diptera: Tachinidae) (Qiao 2007, Yang et al. 2008, Li et al. 2013, Song et al. 2016, Sun et al. 2021).

At present, there have been some studies on the biological control of *H. cunea*, such as studies on the utilization of *C. cunea* (Yang and Zhang 2007), which have achieved remarkable results. However, the damage of *H. cunea* is still serious in China. We need to tap more effective natural enemies like *C. cunea* and clarify their control effects on *H. cunea*. This study systematically explored the populations and community characteristics of natural enemies of *H. cunea* in Dandong to further clarify the dynamics of natural enemy species, coevolution with the host in the invaded habitat of *H. cunea*, and development of biological control technologies.

## Materials and Methods

### Survey Site

**Fig. 1.**
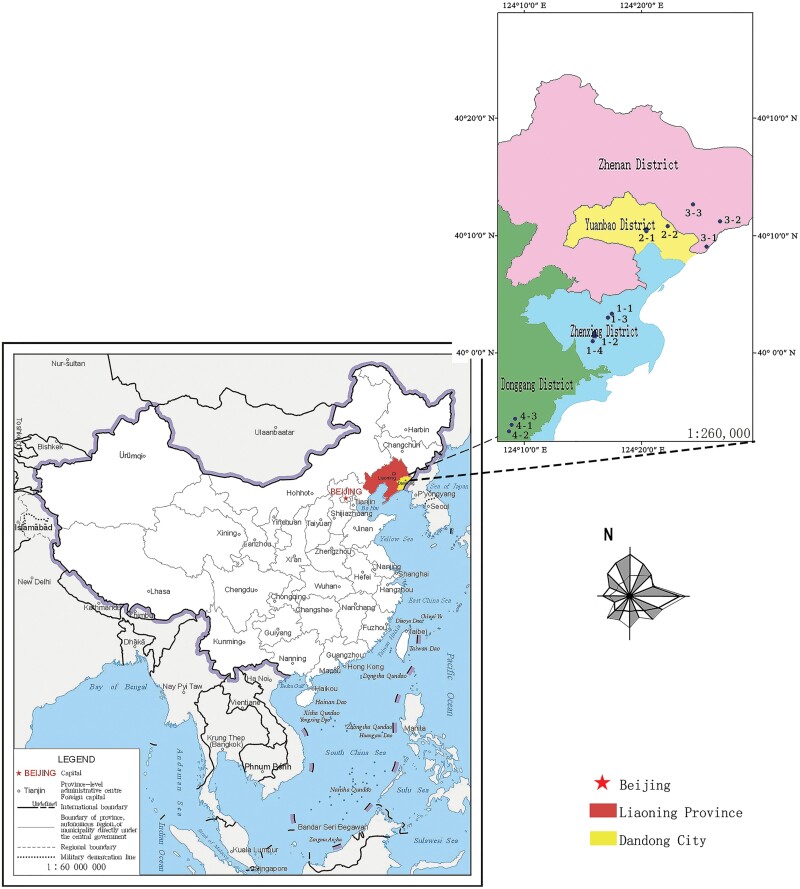
Location of survey sites and zoning map of Dandong, Liaoning Province, China.

The 12 sites were set up in 4 districts (Fig. 1) in Dandong, Liaoning Province: Yuanbao District, Zhen’an District, Zhenxing District, and Donggang District (Table 1).

**Table 1.** Survey sites of *Hyphantria cunea* in Dandong, Liaoning Province, China

District	Site	Latitude (N)	Longitude (E)	Location no.
Zhenxing District	Sunshine Kindergarten	40.0554686°	124.2911985°	1-1
	School of Geological Engineering	40.0274845°	124.2698080°	1-2
	Jielishu village	40.0503457°	124.2852974°	1-3
	Yangzipao village	40.0166442°	124.2637448°	1-4
Yuanbao District	Hamatang	40.1724736°	124.3338970°	2-1
	Zongyucheng	40.1802525°	124.3702957°	2-2
Zhen’an District	Eastern Liaoning University	40.1507432°	124.4254811°	3-1
	Longtouqian village	40.1870491°	124.4444371°	3-2
	Ma jia village	40.2109894°	124.4061186°	3-3
Donggang District	Tufangnan village	39.8976702°	124.1487142°	4-1
	Donggang No.2 Middle School	39.8887153°	124.1450069°	4-2
	Tufangbei	39.9065310°	124.1539026°	4-3

### Investigation Method

The natural enemies of eggs, larvae, and pupae of *H. cunea* on host trees with different generations were collected and surveyed at 12 survey sites from June 2019 to October 2020. At each site, we randomly selected 50 trees and investigated the eggs, webs, and pupae of *H. cunea* on the trees according to local phenology and occurrence of the year. The species and quantity of host trees were recorded in detail. The species and quantity of predators around eggs, webs, larvae, and pupae of *H. cunea* were observed and recorded by taking photographs and videos.

For parasitoids, different developmental stages of *H. cunea* were collected at each survey site. The eggs of *H. cunea* were brought back to the laboratory and placed in a climate chamber at 26 ± 0.5 °C, L:D = 14:10, and 50 ± 5% RH. The larvae were investigated in 2 periods: (i) Larval stage with a web: 3–5 webs were collected for each survey site; (ii) Larval stage without a web: a total of 10–20 mature larvae of *H. cunea* were collected from 3 host trees. The method of pupae collection was the same as that of larvae without a web. All samples were brought back to the laboratory and placed into the incubator at 25 ± 1 °C and fed an artificial diet. Information on the collection time, collection site, and host species was recorded. The species and quantity of parasitoids were observed and recorded every day. All species of natural enemies of *H. cunea* were identified morphologically by consulting experts or reviewing literature and as voucher specimens keep in the Forest Protection Laboratory of Shenyang Agricultural University. The identification of Ichneumonidae was assisted by researcher Maoling Sheng, and the identification of Braconidae, Tachinidae, and Chalcidoidea was assisted by researcher Yanxia Yao (Zhao 1987, Li 2004, He and Chen 2006, He 2015, Yang et al. 2015, Sheng et al. 2016, Zhang and Wang 2017, Cao et al. 2018, Shi et al. 2020).

### Statistical Analysis

The species richness (*S*), importance value index, Shannon–Wiener diversity index (*H’*), and Pielou evenness index (*E*) of the natural enemy community of *H. cunea* were calculated, and principal component analysis (PCA) was carried out. Specific research methods mainly refer to Chen et al. (2020). All statistical analyses were performed using Excel 2010 (Microsoft Corp., Redmond, WA, USA) and SPSS 22.0 (IBM Corp., Armonk, NY, USA) software.

### Evaluation of the Dominant Species of the Natural Enemy Community

The dominant population was determined from the important values index of each species with the formula:


$$\begin{array}{l} Importance\;value\;index=relative\;density+relative\;frequency \end{array}$$

$$\begin{array}{lll} Relative\;density\left(\%\right)=\; & number\;of\;individuals\;of\;a\;certain\;species/\; & number\;of\;all\;individuals\times 100\% \end{array}$$

$$\begin{array}{lll} Relative\;frequency\left(\%\right)=\; & occurrence\;frequency\;of\;one\;species/\; & occurrence\;frequency\;of\;all\;species\times 100\% \end{array}$$

### Diversity and Uniformity of the Natural Enemy Community

The Shannon–Wiener information diversity index (*Hʹ*) was used to calculate the information diversity of each generation of the natural enemy community with the formula:


$$\begin{array}{l} \begin{array}{llll} & H^{\prime }=-\sum Pi\ln Pi,(Pi={}^{Ni}/{}_{N})\; & \; & \; \end{array} \end{array}$$

The Pielou evenness index (*E*) was used to calculate the evenness of each generation, insect period and overall natural enemy community using the formula:


$$\begin{array}{l} E=H^{\prime }/H^{\prime }\max=H^{\prime }/\ln S \end{array}$$

In the formula, *N*_*i*_ is the number of individuals belonging to the species; *N* is the total number of individuals of each species; and *P*_*i*_ is the proportion of all individuals belonging to the species. *Hʹ*_max_ is the maximum value of *Hʹ*; *S* is the number of species.

### PCA of the Natural Enemy Community

SPSS 22.0 statistical software was used for PCA. With the number of the individual species as variables, the feature vectors of each factor were calculated, and the evaluation function was constructed based on the analysis results. The composite degree of the natural enemy community in different periods was comprehensively evaluated and ranked to determine the size and composite degree of the natural enemy community of *H. cunea*.

## Results

### The Investigated Host Trees of *H. cunea*

A total of 16 host plant species of *H. cunea* were found in 12 survey sites within 4 districts in Dandong. These included the following: *Salix* spp., *Ulmus pumila, Begonia evansiana, Rhus typhina, Catalpa ovata, Malus pumila, Pyrus* spp*., Fraxinus mandshurica, Platanus acerifolia, Acer mono, Forsythia suspensa, Morus* spp.*, Populus* spp*., Armeniaca vulgaris, Sophora japonica, Paulownia fortunei* were found in 12 survey sites within 4 districts in Dandong. Survey sites and host tree species are shown in Table 2.

**Table 2.** Host plants of *Hyphantria cunea* at various survey sites in Dandong, Liaoning Province, China

Survey site	Family	Host plant
Donggang District	Salicaceae	*Salix* spp.
	Ulmaceae	*Ulmus pumila*
	Moraceae	*Morus* spp.
	Begoniaceae	*Begonia evansiana*
	Anacardiaceae	*Rhus typhina*
	Bignoniaceae	*Catalpa ovata*
	Rosaceae	*Malus pumila*
	Rosaceae	*Pyrus* spp.
	Oleaceae	*Fraxinus mandshurica*
	Platanaceae	*Platanus acerifolia*
	Salicaceae	*Populus* spp.
Zhenxing District	Salicaceae	*Salix* spp.
	Ulmaceae	*U. pumila*
	Moraceae	*Morus* spp.
	Begoniaceae	*B. evansiana*
	Anacardiaceae	*R. typhina*
	Aceraceae	*Acer mono*
	Oleaceae	*Forsythia suspensa*
	Bignoniaceae	*C. ovata*
	Salicaceae	*Populus* spp.
Yuanbao District	Salicaceae	*Salix* spp.
	Ulmaceae	*U. pumila*
	Moraceae	*Morus* spp.
	Begoniaceae	*B. evansiana*
	Salicaceae	*Populus* spp.
	Platanaceae	*P. acerifolia*
	Rosaceae	*Armeniaca vulgaris*
	Oleaceae	*F. mandshurica*
	Fabaceae	*Sophora japonica*
	Salicaceae	*Populus* spp.
Zhen’an District	Salicaceae	*Salix* spp.
	Ulmaceae	*U. pumila*
	Begoniaceae	*B. evansiana*
	Paulowniaceae	*Paulownia fortunei*
	Rosaceae	*A. vulgaris*
	Salicaceae	*Populus* spp.

### The Natural Enemies of *H. cunea*

A total of 135 predatory natural enemies were collected from 16 host trees of *H. cunea*, and 6,838 parasitic natural enemies emerged. A total of 34 species were identified, from which 20 predators (15 spiders and 5 insects, including *H. axyridis*, *C. shansiensis*, *P. cavipennis*, Labiduridae sp., *A. chinensis*, *Xysticus ephippiafus* Simon (Araneae: Thomisidae)*, Ebrechtella tricuspidata* Fahricius (Araneae: Thomisidae), Clubionidae sp. 1, Clubionidae sp. 2, Clubionidae sp. 3, Tetragnathidae sp., Salticidae sp. 1, Salticidae sp. 2, *Chrysso* sp. (Araneae: Theridiidae), *Misumenops tricuspidatus* Fahricius (Araneae: Thomisidae), Agelenidae sp. 1, *Argiope bruennichi* Scopoli (Araneae: Phalangidae), Thomisidae sp. 1, Araneidae sp., Agelenidae sp. 2), and 14 parasitic natural enemies (10 parasitic wasps and 4 parasitic flies), including *C. gregalis*, *D. singularis*, *P. pupariae*, *P. disparis*, *Trichomalopsis* sp. (Hymenoptera: Pteromalidae), *P. pupariae*, *Barichneumon* sp. (Hymenoptera: Ichneumonidae), *Sympiesis* sp., *Eurytoma* sp. (Hymenoptera: Eulophidae), Cynipoidea sp. (Hymenoptera: Cynipoidea), *C. concinnata*, Tachinidae sp. (Diptera: Tachinidae)), *E. japonica* and *E. fasciata* were identified based on the references (Table 3, see Supplementary Figs. S1 and S2).

**Table 3.** Natural enemies of *Hyphantria cunea* in Dandong, Liaoning Province, China

Type of natural enemy	Species	Stage of *Hyphantria cunea* attacked	Frequency of occurrence in district^a^
Predatory	*Harmonia axyridis*	Egg	ZX 4; DG 1
	*Chrysoperla carnea*	Larva	DG 2
	*Parena cavipennis*	Larva	YB 7; ZX 6; ZA 3
	Labiduridae sp.	Larva	ZX 3; YB 1
	*Arma chinensis*	Larva	ZX 16; YB 3; ZA 2; DG 1
	*Xysticus ephippiafus*	Larva	YB 1; DG 1
	*Ebrechtella tricuspidata*	Larva	ZX 2
	Clubionidae sp. 1	Larva	DG 11; ZA 4; ZX 3; YB 2
	Clubionidae sp. 2	Larva	ZX 1
	Tetragnathidae sp.	Larva	YB 1
	Salticidae sp. 1	Larva	ZX 10
	*Chrysso* sp.	Larva	YB 1
	*Misumenops tricuspidatus*	Larva	ZX 2
	Agelenidae sp. 1	Larva	ZX 2; DG 2
	*Argiope bruennichi*	Larva	YB 1
	Thomisidae sp. 1	Larva	DG 6; ZX 3; YB 2; ZA 1
	Clubionidae sp. 3	Larva	DG 1
	Araneidae sp.	Larva	DG 1
	Salticidae sp. 2	Larva	ZX 1; DG 1
	Agelenidae sp. 2	Larva	ZX 1
Parasitism	*Cotesia gregalis*	Pupa	DG 14; ZX 13
	*Dolichogenidea singularis*	Pupa	DG 7; YB 5; ZX 4; ZA 2
	*Pediobius pupariae*	Pupa	YB 9; ZX 3
	*Pimpla disparis*	Pupa	ZX 15; YB 5
	*Trichomalopsis* sp.	Pupa	YB 1; ZX 1; DG 1
	*Chouioia cunea*	Pupa	YB 46; ZX 9; DG 3
	*Barichneumon* sp.	Pupa	YB 1
	*Sympiesis* sp.	Pupa	DG 1
	*Eurytoma* sp.	Pupa	DG 1
	Cynipoidea sp.	Pupa	ZX 1
	*Compsilura concinnata*	Pupa	ZX 3; YB 1; DG 1
	Tachinidae sp.	Pupa	DG 1
	*Exorista japonica*	Pupa	YB 4; ZX 2
	*Exorista fasciata*	Pupa	DG 1

^a^Note: ZX: Zhenxing District, ZA: Zhen’an District, YB: Yuanbao District, DG: Donggang District.

### Evaluation of the Dominant Species in the Natural Enemy Community of *H. cunea*

The natural enemy community of *H. cunea* was evaluated by the importance value index based on the relative density and relative frequency. The results are as follows:

The ranking of the dominant parasitoids in the natural enemy community was *H. cunea*: *P. pupariae* > *C. cunea > C. gregalis*. The ranking of the dominant predator species was Clubionidae sp. 1 > *A. chinensis > P. cavipennis.* The ranking of dominant species of the natural enemy community of *H. cunea* was *P. pupariae* > *C. cunea > C. gregalis,* which was similar to the results for the parasites (Table 4).

**Table 4.** Ranking of the dominant species of the natural enemy community of *Hyphantria cunea* in Dandong, Liaoning Province, China

Natural enemy species	Frequency	Individuals	Relative density	Relative frequency	Important value index	Rank of dominant species
*Pediobius pupariae*	58	3,807	54.60	22.14	76.73	1
*Chouioia cunea*	12	2,532	36.31	4.58	40.89	2
*Cotesia gregalis*	27	331	4.75	10.31	15.05	3
Clubionidae sp. 1	20	22	0.32	7.63	7.95	4
*Pimpla disparis*	20	20	0.29	7.63	7.92	5
*Arma chinensis*	19	39	0.56	7.25	7.81	6
*Dolichogenidea singularis*	18	18	0.26	6.87	7.13	7
*Parena cavipennis*	16	16	0.23	6.11	6.34	8
Thomisidae sp. 1	12	14	0.20	4.58	4.78	9
Salticidae sp. 1	10	14	0.20	3.82	4.02	10
*Trichomalopsis* sp.	3	94	1.35	1.15	2.49	11
*Exorista japonica*	6	6	0.09	2.29	2.38	12
*Compsilura concinnata*	5	5	0.07	1.91	1.98	13
*Harmonia axyridis*	5	5	0.07	1.91	1.98	13
Labiduridae sp.	4	4	0.06	1.53	1.58	14
Agelenidae sp. 1	4	4	0.064	1.53	1.58	14
*Chrysoperla carnea*	2	2	0.03	0.76	0.79	15
*Xysticus ephippiafus*	2	2	0.03	0.76	0.79	15
*Ebrechtella tricuspidata*	2	2	0.03	0.76	0.79	15
Salticidae sp. 2	2	2	0.03	0.76	0.79	15
*Misumenops tricuspidatus*	2	2	0.03	0.76	0.79	15
*Sympiesis* sp.	1	18	0.26	0.38	0.64	16
*Eurytoma* sp.	1	3	0.04	0.38	0.42	17
*Barichneumon* sp.	1	1	0.01	0.38	0.40	18
Cynipoidea sp.	1	1	0.01	0.38	0.40	18
Tachinidae sp.	1	1	0.01	0.38	0.40	18
*Exorista fasciata*	1	1	0.01	0.38	0.40	18
Clubionidae sp. 2	1	1	0.01	0.38	0.40	18
Tetragnathidae sp.	1	1	0.01	0.38	0.40	18
*Chrysso* sp.	1	1	0.01	0.38	0.40	18
*Argiope bruennichi*	1	1	0.01	0.38	0.40	18
Clubionidae sp. 3	1	1	0.01	0.38	0.40	18
Araneidae sp.	1	1	0.01	0.38	0.40	18
Agelenidae sp. 2	1	1	0.01	0.38	0.40	18
	262	6973	100	100	200	

### Diversity and Evenness of the Natural Enemy Community of *H. cunea*

The Shannon–Wiener information diversity index (*H’*) and Pielou evenness index (*E*) of the natural enemy community of *H. cunea* were calculated based on the data for the parasitic and predatory natural enemies in each developmental stage. It can be seen that the species of natural enemies of larvae were the most abundant (12 species) and differed slightly between different years and generations. There were 9 species of natural enemies of pupae, and the species and quantity of natural enemies for the second generation of pupae were higher than those for the first generation of pupae in the same year. Conversely, there were fewer species of natural enemies of eggs, with only one species being dominant, *Harmonia axyridis* (Table 5).

**Table 5.** Community structure parameters of the natural enemy community of different stages of *Hyphantria cunea*

Developmental stage^a^	Species number (*S*)	Individual number (*N*)	Diversity index (*Hʹ*)	Evenness (*E*)
Gen. 1 of eggs (2019.06.24)	1	2	0	0
Gen. 1 of larvae (2019.07.08)	10	75	1.770	0.768
Gen. 1 of pupae (2019.07.30)	0	0	0	0
Gen. 2 of eggs (2019.08.12)	0	0	0	0
Gen. 2 of larvae (2019.08.19)	10	170	0.850	0.369
Gen. 2 of pupae (2019.11.02)	7	2,144	0.179	0.092
Gen. 1 of eggs (2020.06.20)	1	2	0	0
Gen. 1 of larvae (2020.07.01)	12	199	0.899	0.362
Gen. 1 of pupae (2020.07.21)	0	0	0	0
Gen. 2 of eggs (2020.08.01)	0	0	0	0
Gen. 2 of larvae (2020.08.29)	11	29	2.055	0.857
Gen. 2 of pupae (2020.10.17)	9	4352	0.455	0.207

^a^Gen. 1: generation 1 of *H. cunea*. Gen. 2: generation 2 of *H. cunea.*

The trends of the 2 indices of the natural enemy community of *H. cunea* were mostly the same, and the peak value occurred for the larvae of each generation of *H. cunea* every year. This value reached its highest in the second generation in 2020, indicating that the natural enemy community had high population diversity and the most uniform community distribution in this period.

### PCA of the Natural Enemy Community of *H. cunea*

According to the results in Table 4, we defined the independent variables *P. pupariae* for *X*_*1*_, *C. cunea* for *X*_*2*_, *C. gregalis* for *X*_*3*_, Clubionidae sp. 1 for *X*_*4*_, *A. chinensis* for *X*_*5*_, and *P. cavipennis* for *X*_*6*_, the nondominant parasitic natural enemy population combined with other parasitic natural enemies for *X*_*7*_, and the nondominant predatory natural enemy population combined with other predatory natural enemies for *X*_*8*_. PCA was performed to obtain feature vectors and cumulative contribution rates (Table 6).

**Table 6.** Total variance explained of obtain feature vectors and cumulative contribution rates in PCA of the natural enemy community of *Hyphantria cunea*

Principal component	Initial eigenvalues			Extraction sums of squared loadings		
	Total	% of Variance	Cumulative	Total	% of Variance	Cumulative
1	3.86	48.27	48.27	3.86	48.27	48.27
2	2.28	28.55	76.8	2.28	28.55	76.82
3	1.06	13.21	90.03	1.06	13.21	90.03
4	0.65	8.09	98.11			
5	0.14	1.734	99.85			
6	0.01	0.09	99.94			
7	0.01	0.07	100			
8	1.11E-16	1.39E-15	100			

The cumulative contribution rate of the first 3 principal components in the community was 90.0%, which represents most of the information in the data. Therefore, based on the loadings in Supplementary Table S1, the top 3 principal components (*Y*_*1*_ − *Y*_*3*_) were as follows:


$$\begin{array}{l} \begin{array}{lllllll} Y_{1}= & -0.051ZX_{1}-0.104ZX_{2}+0.187ZX_{3}\; & +0.251ZX_{4}+0.236ZX_{5}\; & +0.243ZX_{6}-0.079ZX_{7}+0.163ZX_{8}\;\;\;=\; & -0.0001X_{1}-0.0002X_{2}+0.0032X_{3}\; & +0.0879X_{4}+0.0458X_{5}+0.1132X_{6}-0.0029X_{7}\; & +0.0275X_{8}-0.5905\; \end{array} \end{array}$$

$$\begin{array}{l} \begin{array}{llllll} Y_{2}=\; & 0.365ZX_{1}+0.229ZX_{2}+0.031ZX_{3}+0.034ZX_{4}\; & +0.019ZX_{5}+0.027ZX_{6}+0.401ZX_{7}+0.298ZX_{8}\;\;\;= & 0.0003X_{1}+0.0004X_{2}+0.0005X_{3}\; & +0.0119X_{4}+0.0037X_{5}+0.0126X_{6}\; & +0.0140X_{7}+0.0503X_{8}-0.7030\; \end{array} \end{array}$$

$$\begin{array}{l} \begin{array}{llllllll} Y_{3}=\; & -0.483ZX_{1}+0.705ZX_{2}+0.132ZX_{3}\; & +0.12ZX_{4}+0.142ZX_{5}\; & +0.123ZX_{6}+0.231ZX_{7}-0.31ZX_{8}\;\;\;= & -0.0004X_{1}+0.0012X_{2}\; & +0.0023X_{3}+0.0420X_{4}+0.0276X_{5}\; & +0.0537X_{6}+0.0086X_{7}\; & -0.0524X_{8}-1.8148\; \end{array} \end{array}$$

In the first principal component *Y*_*1*_, the absolute values of *X*_*4*_ and *X*_*6*_ were higher, indicating that the contribution of Clubionidae sp. 1 and *P. cavipennis* was higher and that they were the main factors affecting the natural enemy community. In the second principal component *Y*_*2*_, the absolute value of *X*_*8*_ was the largest, indicating that the number of other predatory natural enemies was the main factor affecting the community. The absolute values of *X*_*4*_, *X*_*6*_, and *X*_*7*_ were similar, indicating that the effects of Clubionidae sp. 1, *P. cavipennis*, and other parasitic natural enemies on the natural enemy community were also similar. In the third principal component *Y*_*3*_, the absolute values of *X*_*6*_ and *X*_*8*_ were higher, which indicates that Clubionidae sp. 1 and other predators contributed more to the complexity of natural enemies. Finally, the evaluation function was used to evaluate and rank the developmental stages of the natural enemy community (Table 7).

**Table 7.** The ordination of the comprehensive assessment of the developmental stages of the natural enemy community of *Hyphantria cunea*

Stage^a^	Comprehensive scores	Comprehensive ordination	Standard error
Gen. 1 of larvae (2020.07.01)	2.05	1	3.46
Gen. 1 of larvae (2019.07.08)	1.72	2	
Gen. 2 of larvae (2020.08.29)	1.00	3	
Gen. 2 of larvae (2019.08.19)	0.97	4	
Gen. 2 of pupae (2020.10.17)	0.37	5	
Gen. 2 of pupae (2019.11.02)	−0.28	6	
Gen. 1 of eggs (2019.06.24)	−0.91	7	
Gen. 1 of eggs (2020.06.20)	−0.91	7	
Gen. 1 of pupae (2019.07.30)	−1.00	8	
Gen. 2 of eggs (2019.08.12)	−1.00	8	
Gen. 1 of pupae (2020.07.21)	−1.00	8	
Gen. 2 of eggs (2020.08.01)	−1.00	8	

^a^Gen. 1: generation 1 of *H. cunea*. Gen. 2: generation 2 of *H. cunea*.

The evaluation function constructed by PCA was as follows:


$$\begin{array}{l} \begin{array}{lllll} F= & \;0.4827Y_{1}+0.2855Y_{2}+0.1321Y_{3}\;\;=\; & -0.00002X_{1}+0.00018X_{2}+0.00199X_{3}\; & +0.05137X_{4}+0.02681X_{5}+0.06533X_{6}\; & +0.00373X_{7}+0.02877X_{8}-0.72548\; \end{array} \end{array}$$

According to the comprehensive evaluation and ranking of the natural enemy community of *H. cunea*, the comprehensive ranking of the first-generation larvae of *H. cunea* in 2020 was first, while that for the first-generation larvae in 2019 was second, the second-generation larvae in 2020 was third, and the second-generation larvae in 2019 was fourth, which indicates that the larval stage had the highest degree complexity for the natural enemy community of *H. cunea.* The development of the natural enemy community can be divided into 3 stages. The egg is the first stage, the larva is the peak stage, and the pupa is the last stage of development, which indicates that the development of the natural enemy community in *H. cunea* has a certain regularity.

## Discussion

Parasitism of dominant parasitic species in *H. cunea.* The dominant parasitic species of *H. cunea* in Dandong were *P. pupariae*, *C. cunea*, and *C. gregalis*, which was similar to those in Shenyang (Chen et al. 2020). *Trichomalopsis* sp., *Eurytoma* sp., and *Sympiesis* sp. were found in Dandong but were not reported in Shenyang. The different geographical locations, altitudes, climates, and other conditions might be the reasons for the different natural enemy components, reflecting not only the species of natural enemies but also their quantity and parasitic abilities. Chinese scholars have studied the parasitism rate of *H. cunea*; 8.41% of *P. pupariae* was found on overwintering pupae in Qinhuangdao, Hebei Province, whereas only 1.84% was found in Shenyang, Liaoning Province (Qiao 2007, Wang et al. 2010). Likewise, 13.25%–13.4% of *C. cunea* was found in Hebei and Shandong, yet 2.60%–8.94% was found in Liaoning (Yue et al. 2016). The average parasitism rate of *P. disparis* on summer pupae in Dalian, Liaoning Province, was 9.84%, which was higher than that in Hebei and Shandong (0.33%–2.01%) (Yang et al. 2001, Qu et al. 2006).

In recent years, studies on the utilization of *C. cunea* have achieved remarkable results, but there are few relatively basic studies on the other natural enemies of *H. cunea* (Yang and Zhang 2007). Consequently, further studies on the biological characteristics, host specialization, and artificial breeding of other dominant populations of the natural enemy community, that is, those of *P. pupariae* and *C. gregalis*, should be carried out.

In addition, we observed an phenomenon regarding mixed parasitism: *P. pupariae* and *C. cunea* parasitized the same pupae of *H. cunea*. In this case the number of emergence in *C. cunea* was significantly reduced, the body size was smaller and the survival rate was lower than that under polyparasitism. The interspecific competitiveness of *C. cunea* might be the reason for this phenomenon. It will be helpful to clarify the dominant species at the pupal stage and provide a theoretical basis for the biocontrol of *H. cunea*.

Predation of dominant predatory species in *H. cunea.* The dominant predatory natural enemies in Dandong are Clubionidae sp. 1, *A. chinensis* and *P. cavipennis.* Among them, *P. cavipennis* has strong adaptability and fecundity and is an excellent predator (Chen et al. 2020). Its adults and larvae can feed on common lepidopteran insects such as *Phalera fuscescens* Butler (Lepidoptera: Notodontidae), *P. birnicola* Bryk (Lepidoptera: Notodontidae), *Ivela ochropoda* Eversmann (Lepidoptera: Lymantriidae), *Epicopeia mencia* Moore (Lepidoptera: Epicopeiidae), and *Antheraeae pernyi* Guerin-Meneville (Lepidoptera: Saturniidae) (Wang et al. 1992, Zhao et al. 2011). In the investigation, we found Clubionidae sp. 1 in all 4 regions and believe it will be effective against *H. cunea* in Dandong with good prospects for utilization in the future. However, the current study on Clubionidae focuses on comparative morphological research (Zhang 2018, Li 2019, Chen 2020), but its predation ability has not been reported.

In the study, most of the spiders we collected were the safari type, which hunt prey and do not weave webs or build tents (Zhang and Wang 2017, Xing and Liang 2019). In contrast, netting spiders attach their webs to the prey, which was beneficial to its predation of the larvae of *H. cunea*, that is, *A. difficilis* attaches webs to the surface of eggs to prey on the early-instar larvae of *H. cunea* (Shu and Yu 1985). Spiders are one of the most important predators of *H. cunea* in the field, but their predatory ability remains underexplored. Further, species-level identification of some spiders, other predators, and parasitoids of *H. cunea* is needed.

*Suggestions and prospects*. The complexity of the natural enemy community in Dandong was higher in the larval stage than in the egg and pupal stages, which was consistent with the findings in Shenyang (Nan et al. 2019). A similar situation was also observed in other forestry pests; for example, *Monema flavescens* Walker (Lepidoptera: Limacodidae) has abundant natural enemies in the egg and larval stages (Wang et al. 1993), and *Dendrolimus superans* Butler (Lepidoptera: Lasiocampidae) has many natural enemies in the larval and pupal stages (Wang et al. 1996). The larval stage is a developmental stage with a high complexity of natural enemies of forestry pests, and it is also the main insect stage that harms host plants. Further study on the dominant natural enemies and biological characteristics of the larval stage can provide a useful reference for the biological control against these pests.

The study of the natural enemy community of *H. cunea* in the invaded habitat will help with further clarifying the phylogeny of natural enemy species groups, coevolution with the host, and developing corresponding biological control technologies. In particular, studying the interaction between members of the natural enemy community and its biological control strategies should be the major direction in the future.

We identified the dominant species groups of natural enemies at invasion sites, explored their biology, ecology, artificial rearing, and release techniques, and evaluated their control effects in field trials (Chen et al. 2022, Zhang et al. 2023). However, there are some things that have not been considered in our experiment. For example, many studies consist of surveys that sample only 1 period of the pest occurrence in the field, or focus only on one enemy, making it difficult to understand the combined effects of complete natural enemy guilds on pest population dynamics (Dainese et al. 2017). In fact, empirical studies indicate that trophic interactions among diverse natural enemy assemblages may result in a full spectrum of outcomes including null, additive, antagonistic, or synergistic effects (Letourneau et al. 2009).

The utilization of other natural enemies of *H. cunea* has rarely been reported except for parasitoids. According to our results, we should strengthen the research on the application of predatory natural enemies, including spiders, for instance, how do spiders prey on the fall webworm? What are their biological and ecological characteristics? Can they be reared? How do they affect other natural enemies with increases in their populations in the environment? These issues need to be further studied and solved to provide new paths and solutions for the biological control of *H. cunea.*

## Supplementary Material

iead105_suppl_Supplementary_Figures

iead105_suppl_Supplementary_Tables_S1
