# Supplementary material for: Dynamics of the natural enemy community of Hyphantria cunea (Lepidoptera: Erebidae) in Dandong, China
Source: J Insect Sci. 2023 Nov 28;23(6):8. doi: 10.1093/jisesa/iead105 (PMC10684046; doi:10.1093/jisesa/iead105)
Supplement: iead105_suppl_Supplementary_Tables_S1 [file iead105_suppl_supplementary_tables_s1.docx]

Supplementary Table S1. Eigenvectors of the natural enemy community

|  | Component | | |
| --- | --- | --- | --- |
|  | 1 | 2 | 3 |
| *Pediobius pupariae* = *X_1_* | -0.051 | 0.365 | -0.483 |
| *Chouioia cunea* = *X_2_* | -0.104 | 0.229 | 0.705 |
| *Cotesia gregalis* = *X_3_* | 0.187 | 0.031 | 0.132 |
| Tachinidae sp. *= X_4_* | 0.251 | 0.034 | 0.12 |
| *Arma chinensis* = *X_5_* | 0.236 | 0.019 | 0.142 |
| *Parena cavipennis* = *X_6_* | 0.243 | 0.027 | 0.123 |
| Other parasitic natural enemies = *X_7_* | -0.079 | 0.401 | 0.231 |
| Other predatory natural enemies = *X_8_* | 0.163 | 0.298 | -0.31 |
